# miR-21 Expression Determines the Early Vaccine Immunity Induced by *LdCen*^−/−^ Immunization

**DOI:** 10.3389/fimmu.2019.02273

**Published:** 2019-09-24

**Authors:** Sreenivas Gannavaram, Parna Bhattacharya, Abid Siddiqui, Nevien Ismail, Subha Madhavan, Hira L. Nakhasi

**Affiliations:** ^1^Division of Emerging and Transfusion Transmitted Diseases, Center for Biologics Evaluation and Research, US Food and Drug Administration, Silver Spring, MD, United States; ^2^Innovation Center for Biomedical Informatics, Georgetown University, Washington, DC, United States

**Keywords:** leishmaniasis, microRNA, miR-21, IL-12, live attenuated *Leishmania* vaccines, biomarker, parasitic vaccines

## Abstract

No vaccine exists against visceral leishmaniasis. Toward developing vaccines against VL, we have reported previously on the immunogenicity of live attenuated *LdCen*^−/−^ parasites in animal models. Immunization with *LdCen*^−/–^ parasites has been shown to induce durable protective immunity in pre-clinical animal models. Although the innate immune responses favoring a Th1 type immunity are produced following *LdCen*^−/−^ immunization, the molecular determinants of such responses remain unknown. To identify early biomarkers of immunogenicity associated with live attenuated parasitic vaccines, we infected macrophages derived from healthy human blood donors with *LdCen*^−/−^ or *LdWT* parasites *ex vivo* and compared the early gene expression profiles. In addition to altered expression of immune related genes, we identified several microRNAs that regulate important cytokine genes, significantly altered in *LdCen*^−/–^ infection compared to *LdWT* infection. Importantly, we found that *LdCen*^−/–^ infection suppresses the expression of microRNA-21 (miR-21) in human macrophages, which negatively regulates IL12, compared to *LdWT* infection. In murine DC experiments, *LdCen*^−/−^ infection showed a reduced miR-21 expression with a concomitant induction of IL12. Silencing of miR-21 using specific inhibitors resulted in an augmented induction of IL12 in *LdWT* infected BMDCs, illustrating the role of miR-21 in *LdWT* mediated suppression of IL12. Further, exosomes isolated from *LdCen*^−/−^ infected DCs contained significantly reduced levels of miR-21 compared to *LdWT* infection, that promoted proliferation of CD4^+^ T cells *in vitro*. Similar miR-21 mediated IL12 regulation was also observed in *ex vivo* human macrophage infection experiments indicating that miR-21 plays a role in early IL12 mediated immunity. Our studies demonstrate that *LdCen*^−/−^ infection suppresses miR-21 expression, enables IL12 mediated induction of adaptive immunity including proliferation of antigen experienced CD4^+^ T cells and development of a Th1 immunity, and suggest that miR-21 could be an important biomarker for *LdCen*^−/−^ vaccine immunity in human clinical trials.

**One Sentence Summary:**

Role of miR-21 in vaccine induced immunity.

## Introduction

Leishmaniasis, caused by the protozoan parasites *Leishmania*, is a neglected protozoan disease that mainly affects the tropical as well as subtropical countries of the world. Annually, 200,000–400,000 new cases of visceral leishmaniasis (VL) are reported that results in 20,000–40,000 deaths with 95% of the fatal cases occurring in six countries, namely, India, Bangladesh, Sudan, Nepal, Ethiopia, and Brazil (1). Globally, 0.7–1.2 million new cases of cutaneous leishmaniasis (CL) occur every year (2). Despite the recent advances in antileishmanial chemotherapeutics, eradication of the disease by chemotherapy alone is not possible (3). Since *Leishmaniasis* is a disease of the poor occurring mostly in remote rural villages with poor housing and little access to modern health-care, chemotherapy may be inadequate (4). Currently, there are no FDA-licensed vaccines or screening tests for *Leishmania* parasites in the US. Immigration to US from Latin American, African, and Middle Eastern countries, areas with high incidence of Leishmaniasis, is increasing. Although most of the cases of leishmaniasis diagnosed in the United States are in people who became infected while traveling or living in other countries, autochthonous cases of cutaneous leishmaniasis are reported in southern United States [https://www.cdc.gov/parasites/leishmaniasis/gen_info/faqs.html, (5)]. These factors pose a serious risk of spread of the disease in the US by these blood-borne pathogens. Prevalence of canine leishmaniasis and reports of vector transmission of *Leishmania* parasites in southern United States further highlight the potential for widespread dissemination (6). Therefore, there is an urgent need for developing effective vaccines against human leishmaniasis.

We have reported previously on the protective efficacy induced by centrin gene deleted *Leishmania donovani* parasites (*LdCen*^−/−^) as live attenuated vaccines (7–10). The protective immunity induced by *LdCen*^−/−^ parasites was mediated by strong multifunctional CD4^+^ and CD8^+^ T cell responses (8). Further studies showed that immunization with *LdCen*^−/−^ parasites induces strong pro-inflammatory response including IL-12, IFN-γ, IL-17, and reduced IL-10 production from macrophages compared to virulent wild type *L. donovani* (*LdWT*) infection (11, 12). Studies showed that following immunization, *LdCen*^−/−^ parasites alter the early innate immune response including programming of parasitized macrophages to M1 type, increased expression of co-stimulatory signals, subdued co-inhibitory signals on the parasitized dendritic cells, and secretion of pro-inflammatory cytokines compared to virulent parasite infection (13). However, the molecular determinants of early innate immune responses following immunization remain to be determined. Identification of the molecular determinants of early immune response not only reveals the immune mechanisms important for protective immunity but also lead to biomarkers of protection that would aid in advancing the vaccines toward clinical studies and regulatory approvals.

MicroRNAs (miRNAs) are noncoding ssRNAs of 19–25 nucleotide in length that mediate post-transcriptional regulation of various target genes (14, 15). miRNAs usually bind to sites of nucleotide sequence complementarity in the 3-untranslated region (UTR) of target genes and regulate their gene expression by mechanisms including inhibition of translation, degradation of mRNA (16). miRNAs play critical roles in a broad range of biological processes, including development, stress response, cancer, and cardiac hypertrophy therefore the roles of miRNAs in both normal homeostatic and pathological processes are widely studied (17, 18). Due to the important roles played by microRNAs, their role in innate immunity is investigated with great interest (19, 20). More specifically miR-21 has been shown to be a negative regulator of IL-12 in allergic inflammation (21). In this study we have determined the microRNAs whose expression is specifically altered following *Leishmania* infection and investigated the role of miR-21 in the regulation of IL-12 in both murine and *ex vivo* human infection studies in the context of *LdCen*^−/−^ immunization.

## Results

### Distinct microRNA Expression Profiles Are Induced in Human Macrophages Upon *LdCen-/-* Infection Compared to *LdWT* Infection

**Figure 1:**
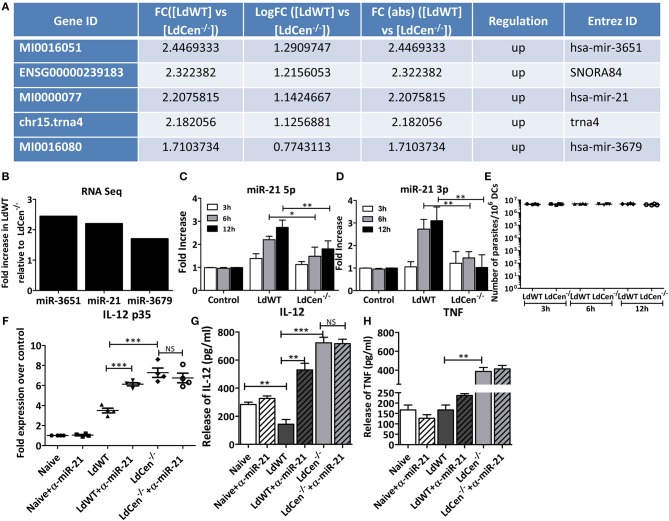
Expression of miR-21 in human macrophages and murine DCs. **(A)** Small RNAseq results showing the enrichment of microRNAs and snoRNAs in *LdWT* infection compared to *LdCen*^−/^^−^ infected human macrophages differentiated *in vitro* from elutriated monocytes. **(B)** Human macrophages, infected with *LdWT* or *LdCen*^−/−^ showing elevated expression of miR-3651, miR21, and miR-3679 in *LdWT* compared to *LdCen*^−/−^ infection. **(C)** Expression of miR-21 5p in murine DCs infected with *LdWT* or *LdCen*^−/^^−^ parasites at 3, 6, and 12 h post infection. **(D)** Expression of miR-21 3p in murine DCs infected with *LdWT* or *LdCen*^−/^^−^ parasites at 3, 6, and 12 h post infection. The fold increase in expression is compared to that observed in naïve DCs. **(E)** qRT-PCR assay showing the parasite burden in the murine DCs infected with *LdWT* or *LdCen*^−/^^−^ parasites at the indicated time points. **(F)** Expression of IL-12p35 mRNA in the murine DCs either in presence or absence of anti-miR-21 5p oligonucleotides delivered through exosomes is measured by qRT-PCR. **(G)** Expression of IL-12 in the culture supernatants of murine DCs infected with *LdWT* or *LdCen*^−/^^−^ parasites 12 h post-infection in presence or absence of anti-miR-21 oligonucleotides by ELISA. **(H)** Expression of TNF protein in the culture supernatants of macrophages infected with *LdWT* or *LdCen*^−/^^−^ parasites measured by ELISA is shown. Statistical significance between miR-21 or IL-12 expression in *LdWT* and *LdCen*^−/^^−^ infections at various time points post infection is shown (*t*-test; ^*^p < 0.05, ^**^p < 0.01, ^***^p < 0.001).

To test whether distinct microRNAs are expressed following infection of macrophages with either *LdWT* or *LdCen*^−/−^ parasites replica macrophage cultures from elutriated human monocytes were established. Monocytes were differentiated *in vitro* into macrophages and infected with *LdWT* or *LdCen*^−/^^−^ parasites. Replica macrophage cultures were stained with Diff-Quik reagent after 24 h infection. The percentage of infected macrophages and the parasite indices were comparable across all infections and donors (Supplementary Figure 1). The quality of RNA was assessed by a RNA Bioanalyzer and RNAseq was performed on the isolated RNA samples. RNA sequencing revealed distinct microRNA expression profiles in human macrophages upon *LdCen*^−/^^−^ infection compared to *LdWT* infection (Figure 1A, Supplementary Figure 2). We performed a pairwise comparison of the expression of small RNAs between uninfected, *LdWT* infected, and *LdCen*^−/^^−^ infected macrophages. Results indicated that several microRNAs are enriched in *LdWT* infection compared to *LdCen*^−/^^−^ infection in healthy donors (*p* < 0.05, Supplementary Table 1, Figure 1B). Of the several microRNAs that are enriched in *LdWT* infection compared to *LdCen*^−/−^ infection, miR-21 was selected for further study due to its role in the regulation of IL-12 expression in recent studies (21, 22).

### Suppression of miR-21 Expression in LdCen-/- Infected Murine DCs *in vitro*

To test whether suppression of miR-21 expression observed in human macrophages due to *LdCen*^−/^^−^ infection can be recapitulated in antigen presenting cells in murine models and thus enable understanding of the role of miR-21 in *LdCen*^−/^^−^ induced innate immunity, we first performed *in vitro* infection experiments with bone marrow derived DCs from C57bl/6 mice. Further DCs have been recognized as the main producer of IL-12 following *Leishmania* infection (23). BMDCs infected with *LdCen*^−/^^−^ parasites induced significantly reduced expression of miR-21 compared to *LdWT*^−^ infection as early as 3 h post infection (Figure 1C, ^*^p < 0.05, ^**^p < 0.01). High induction of miR-21 5p was sustained even at 6 and 12 h post infection in *LdWT* infection compared to *LdCen*^−/^^−^ infection. Both the isoforms of miR-21 i.e., miR-21 5p and miR-21 3p were found to be significantly suppressed in *LdCen*^−/^^−^ infection compared to *LdWT* infection (Figures 1C,D, ^*^p < 0.05, ^**^p < 0.01). The pre-miRNA hairpin is cleaved by the RNase III enzyme Dicer to generate miRNA-5p and miRNA-3p. Among these the 5p strand is present in the forward (5′-3′) position, while the 3p strand is located in the reverse position. RT-PCR assay to measure the *Leishmania* minicircle DNA showed a comparable parasite infection in the DCs *in vitro* across the time points suggesting that the observed differences in miR-21 levels between *LdWT* and *LdCen*^−/−^ are not due to variation in the parasite infection levels (Figure 1E). Since miR-21 has been shown to target 3′UTR of the mRNA encoding IL-12p35 for degradation, we measured the expression of IL-12p35 by qRT-PCR. Results showed that the abundance of IL-12 is inversely proportional to miR-21 expression (Figure 1F, ^***^p < 0.001). Further, blocking of miR-21 using anti-sense oligonucleotides delivered through murine DC exosomes showed restoration of the IL-12 expression in DCs infected with *LdWT*. No significant difference was observed in IL-12p35 expression in murine DCs upon *LdCen*^−/−^ infection treated with anti-miR-21 oligonucleotides due to low level of mR-21 expression observed in *LdCen*^−/−^ infection. However, the IL-12 protein levels measured in the culture supernatants showed an increase upon miR-21 inhibition in *LdWT* infection (Figure 1G, ^**^p < 0.01, ^***^p < 0.001) consistent with the mRNA expression of IL-12 (Figure 1F). We did not observe any significant differences in TNF due to miR-21 inhibition in *LdWT* or *LdCen*^−/^^−^ infections even though significant difference was observed between *LdWT* and *LdCen*^−/^^−^ infections without miR-21 inhibition (Figure 1H, ^**^p < 0.01). Consistent with our previous observations in *LdWT* and *LdCen*^−/^^−^ infections in mice (8), there was a slightly lower but not significant difference in the IL-10 level (*p* = 0.135). Similarly, a slightly higher but not significant IFN-γ level (*p* = 0.058) was observed between *LdWT* and *LdCen*^−/^^−^. No significant differences were observed between untreated and α-miR-21 treated groups in both infection groups suggesting that miR-21 effect is mainly observed in IL-12 expression (Supplementary Figure 2).

### *In vivo* Inhibition of miR-21 Due to *LdCen-/-* Infection Restores IL-12 Expression

**Figure 2:**
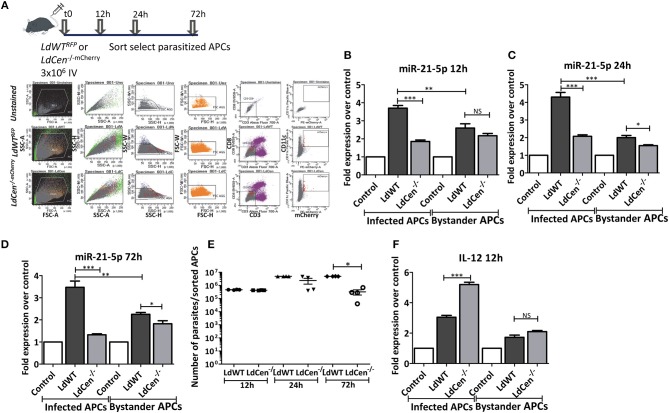
Expression of miR-21 in parasitized murine APCs. **(A)** C57Bl/6 mice were intravenously injected with either *LdWT*^*RFP*^ or *LdCen*^−/^^−mCherry^ recombinant parasites. After 12, 24, or 72 h following infection, parasitized APCs were sort selected from the cell suspensions prepared from spleen and inguinal, axillary, superficial cervical, submandibular, and parotid lymph nodes lymph nodes. The gating scheme for selection of the parasitized APCs is shown. **(B)** Expression of miR-21 5p was measured in parasitized and non-parasitized APCs (Bystander APCs) in *LdWT* and *LdCen*^−/^^−^infection groups is measured at 12, **(C)** 24, and **(D)** 72 h post infection by qRT-PCR. Fold increase in expression of miR-21 is relative to the expression observed in uninfected control DCs. **(E)** qRT-PCR assay showing the parasite burden in the parasitized murine APCs infected with *LdWT* or *LdCen*^−/^^−^ parasites at the indicated time points. **(F)** Expression of IL-12 mRNA in *LdWT* or *LdCen*^−/^^−^ infected and bystander APCs is measured by q-RT-PCR. Statistical significance between miR-21 or IL-12 expression in *LdWT* and *LdCen*^−/^^−^ infections at various time points post infection is shown (*t*-test; ^*^p < 0.05, ^**^p < 0.01, ^***^p < 0.001).

To further confirm our results from the *in vitro* DC infection experiments, we performed miR-21 expression analysis *in vivo* in mice infected with *LdWT* or *LdCen*^−/^^−^ parasites. Infection with either red fluorescence protein expressing *LdWT* (*LdWT*^*RFP*^) or mCherry expressing *LdCen*^−/^^−^ (*LdCen*^−/^^−mCherry^) parasites allowed us to isolate APCs infected with parasites from spleen and lymph nodes for analysis by fluorescence-based sorting (Figure 2A). miR-21 expression in parasitized cells was tested at various time points after infection with either *LdWT* or *LdCen*^−/−^ parasites. Similar to our *in vitro* studies, miR-21 expression was found to be reproducibly high in cells infected with *LdWT* compared to *LdCen*^−/^^−^ infection starting from 12 h post infection to all the time points tested (Figures 2B–D, ^*^p < 0.05, ^**^p < 0.01, ^***^p < 0.001). RT-PCR assay to measure the *Leishmania* minicircle DNA showed a comparable parasite infection in the parasitized APCs *in vivo* at 12 and 24 h time points following infection suggesting that the observed differences in miR-21 levels between *LdWT* and *LdCen*^−/−^ are not due to variation in the parasite infection level (Figure 2E, ^*^p < 0.05). As expected, the number of *LdCen*^−/−^ parasites showed a decline at the 72 h time point due to the limited replication as a result of deletion of centrin gene in these parasites (Figure 2E, ^*^p < 0.05). Of interest, we found that miR-21 expression was also altered in non-infected i.e., bystander cells. The bystander cells also showed high expression of miR-21 compared to uninfected controls although it was significantly less than that observed in the parasitized cells at all the time points tested (Figures 2B–D). *LdCen*^−/−^ infection induced consistently less miR-21 compared to *LdWT* infection at all time points tested in parasitized and bystander cells (Figures 2B–D). Measurements of IL-12 mRNA in the parasitized cells showed a reproducible higher expression in *LdCen*^−/^^−^ infection compared to *LdWT* infection, consistent with our *in vitro* results (Figure 2F, ^***^p < 0.001). In contrast, the bystander cells did not show significant variation in IL-12 between *LdWT* and *LdCen*^−/^^−^ infections at 12 h point (Figure 2F).

### Suppression of miR-21 Occurs in DCs Only Upon Live *LdCen-/-* Infection

**Figure 3:**
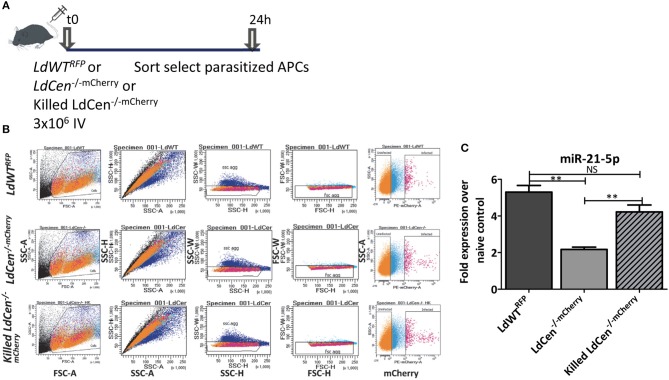
Only live *LdCen*^−/^^−^ parasites can reduce the induction of miR-21 expression in parasitized DCs. **(A)** C57Bl/6 mice were intravenously injected with either live *LdWT*^*RFP*^, live *LdCen*^−/^^−mCherry^, or killed *LdCen*^−/^^−mCherry^ recombinant parasites. After 24 h following infection, parasitized DCs were sort selected from the cell suspensions prepared from spleen and inguinal, axillary, superficial cervical, submandibular, and parotid lymph nodes lymph nodes. **(B)** The gating scheme for selection of the parasitized DCs is shown. **(C)** Expression of miR-21 5p was measured in parasitized in live *LdWT*^*RFP*^, live *LdCen*^−/^^−mCherry^, or killed *LdCen*^−/^^−mCherry^ infection groups is measured at 24 h post infection by qRT-PCR. Fold increase in expression of miR-21 is relative to the expression observed in DCs from uninfected mice. Statistical significance in miR-21 expression in between live *LdWT*^*RFP*^, live *LdCen*^−/^^−mCherry^, or killed *LdCen*^−/^^−mCherry^ infections is shown (*t*-test; ^**^p < 0.01).

Our results indicated that infection with live virulent or live attenuated parasites results in distinctly altered miR-21 expression and impacts the innate and adaptive immunity in significant ways. This led to a hypothesis whether live attenuated parasites are necessary to suppress the miR-21 expression or similar effect could also be achieved by a killed parasite. A killed attenuated parasite would be considered safer vaccine antigen compared to live attenuated parasite. To verify if expression of miR-21 in dendritic cells is dependent on infection with live *LdCen*^−/^^−^ parasites, we infected mice with live *LdWT*^*RFP*^ or *LdCen*^−/^^−mCherry^ or killed *LdCen*^−/^^−mCherry^ parasites and 24 h post infection, parasitized DCs were sort selected from spleen and lymph nodes (Figures 3A,B). In experiments with killed fluorescent *LdCen*^−/^^−^ parasites, since no replication of these parasites occurs *in vivo*, We have selected 24 h post infection to isolate parasitized DCs to assess the expression of miR-21. We did not observe any loss of fluorescence in killed *LdCen*^−/^^−mCherry^ parasites due to the fixing process (Figure 3B, killed *LdCen*^−/^^−mCherry^ panel). Expression of miR-21 was measured by RT-PCR in cells sort selected based on RFP/mCherry fluorescence. Results showed that only infection with viable *LdCen*^−/^^−mCherry^ parasites induced a reduction in miR-21 expression compared to *LdWT*^*RFP*^ infection (Figure 3C, ^**^p < 0.01). No significant reduction in miR-21 occurred in the cells infected with killed *LdCen*^−/^^−mCherry^ parasites compared to *LdWT*^*RFP*^ infection (Figure 3C). These results suggest that alteration of miR-21 expression is dependent on presence of live parasite rather than killed parasite antigen.

### Blocking of *LdWT* Infection Induced miR-21 Restores CD4+ T Cell Proliferation

**Figure 4:**
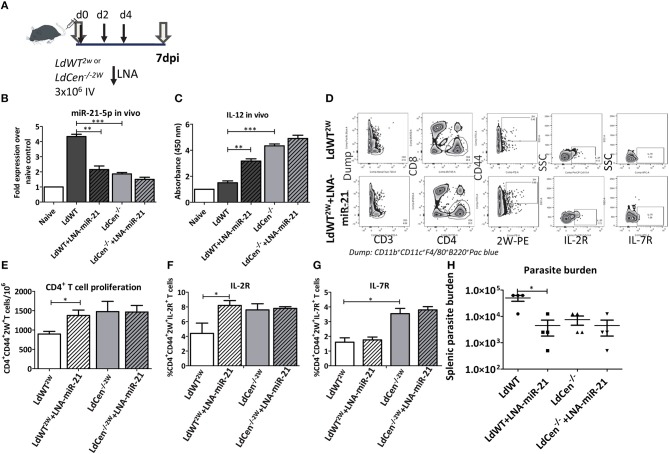
Blocking miR-21 induction rescues IL-12 expression. **(A)** C57Bl/6 mice were infected with recombinant wild type or centrin deleted *Leishmania* parasites secreting CD4^+^ T cell epitope 2W (EAWGALANWAVDSA) fused to *L. donovani* 3′ Nucleotidase/Nuclease protein lacking the N' terminus membrane anchoring domain. The mice were administered with LNA chemistry based anti-miR-21 oligonucleotides on days 0, 2, and 4 following parasite infection. **(B)** Expression of miR-21 5p in the splenocytes 7 days post-infection with *LdWT* or *LdCen*^−/^^−^ parasites in mice treated with anti-miR-21 oligonucleotides. **(C)** Expression of IL-12 in the splenocytes 7 days post-infection with *LdWT* or *LdCen*^−/^^−^ parasites in mice treated with anti-miR21 oligonucleotides. **(D)** Epitope-specific activated CD4^+^ T cells expressing IL-2R and IL7-R markers were identified using flow cytometric analysis 7 days post infection. Gating scheme to identify the activated CD4^+^ T cells expressing surface markers IL-2R, and IL7R from the spleen and lymph nodes of *LdWT*^2*W*^ infected and or LNA-miR-21 treated mice is shown. To enrich 2W epitope specific CD4^+^ T cell populations, 2W tetramers were used. Dump contained markers for B cells (B220), macrophages (F4/80), DCs, and monocytes (CD11c, CD11b). **(E)** Proliferation of 2W epitope specific CD4^+^ T cell population enriched from spleen and lymph nodes in presence or absence of anti-miR-21 oligonucleotides using tetramers is shown. **(F)** Expression of IL-2R on the activated CD4^+^ 2W^+^ T cell population in *LdWT*^2*W*^ or *LdCen*^−/^^−2W^ infected mice indicating the proliferation is shown. **(G)** Expression of early CD4^+^ T cell central memory marker IL-7R on the activated CD4^+^ 2W^+^ T cell population in *LdWT*^2*W*^ or *LdCen*^−/^^−2W^ infected mice is shown. **(H)**. Splenic parasite burden following treatment with LNA-miR-21 in *LdWT* or *LdCen*^−/^^−^ infected mice is shown. Statistical significance between CD4^+^ T cell proliferation and expression of memory markers in *LdWT* and *LdCen*^−/^^−^ infections 7 days post infection is shown (*t*-test; ^*^p < 0.05, ^**^p < 0.01, ^***^p < 0.001).

To determine if miR-21 mediated IL-12 regulation occurs *in vivo*, mice were infected with *LdWT* or *LdCen*^−/−^ parasites and treated with LNA chemistry based anti-miR-21 oligonucleotides on days 0, 2, and 4 (Figure 4A). Splenic DCs from the infected mice were isolated 7 days post-infection and the expression of miR-21 and IL-12 was measured. Results showed that consistent with our previous studies, *LdCen*^−/−^ infection induced reduced levels of miR-21 and correspondingly high levels of IL-12 compared to *LdWT* infection (Figures 4B,C, ^**^p < 0.01, ^***^p < 0.001). Treatment with LNA resulted in a significant reduction of miR-21 in *LdWT* infection (Figure 4B). Similar reduction was not evident in *LdCen*^−/−^ infection presumably due to low induction of miR-21 following infection which was also observed in our *in vitro* studies (Figures 4B,C). Correspondingly the IL-12 expression was significantly higher in LNA treated *LdWT* infection compared to *LdWT* infected control (Figure 4C). Similar increment in the expression of IL-12 was not observed in *LdCen*^−/−^ upon LNA treatment since the background level of IL-12 expression to begin with was higher in *LdCen*^−/−^ compared to *LdWT* infection (Figure 4C). To measure if the altered level of IL-12 in these groups impacts the adaptive immune responses, we infected the mice with *LdWT* and *LdCen*^−/−^ parasites secreting CD4^+^ T cell specific 2W peptide. Using tetramers against 2W peptide, epitope specific CD4^+^ T cells were isolated and identified on flow cytometry (Figure 4D). Results showed that inhibition of miR-21 by LNA-oligonucleotides significantly increases the CD4^+^CD44^+^ 2W^+^ T cell population compared to untreated *LdWT*^2*W*^ infection. Treatment with LNA had no effect in the 2W specific CD4^+^T cell population in *LdCen*^/−2W^ infection (Figure 4E, ^*^p < 0.05). To test the proliferation of 2W specific population following infection with *LdWT* or *LdCen*^−/^^−^ parasite infection, IL-2R expression was used as a marker. *LdCen*^/−2W^ infection resulted in significantly higher IL-2R^+^ CD4^+^ T cells as was observed in our previous studies (8). Consistent with the increased proliferation, IL-2R expression was also significantly higher in LNA treated *LdWT* infection compared to untreated group (Figure 4F, ^*^p < 0.05). Although proliferation of the 2W specific CD4^+^ T cell population was observed in LNA treated *LdWT* infection, expression of other markers of early memory such as IL-7R were absent in this population. In contrast, a significantly higher IL-7R^+^2W^+^CD4^+^ T cell population was observed *LdCen*^−/^^−^ infection similar to our previously published results (24) and no change in expression was observed upon LNA treatment (Figure 4G, ^*^p < 0.05). To measure if blocking miR-21 expression impacts the parasite proliferation, we measured the splenic parasite burden following treatment with LNA treatment. Splenic parasite burden 2 weeks after LNA treatment showed that blocking miR-21 expression significantly reduces the parasite burden in *LdWT* infection (Figure 4H, ^*^p < 0.05).

### Exosomes Released From *LdWT* Infected DCs Contain High Levels of miR-21

**Figure 5:**
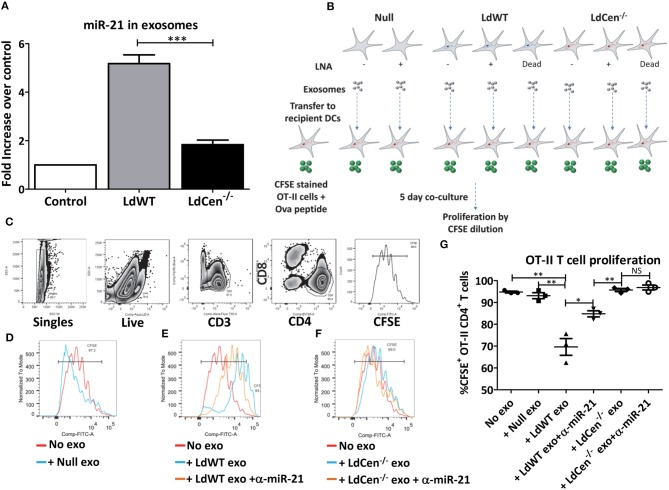
Exosomal miR-21 affects the CD4^+^ T cell proliferation. **(A)** Exosomes from the culture supernatants of murine DCs infected with *LdWT* or *LdCen*^−/^^−^ parasites were purified and the expression of miR-21 was measured in qRT-PCR. Exosomes from uninfected DCs were used as control. Fold increase in miR-21 expression was based on the uninfected control DCs. **(B)** Schematic of the experiment to determine the impact of exosomal miR-21 on the CD4^+^ T cell proliferation. Murine BMDCs were infected with live or dead *LdWT* or *LdCen*^−/^^−^ parasites. DCs were treated with anti-miR-21 LNAs to block the miR-21 as indicated. Exosomes were purified from the infected and or treated DC culture supernatants. The exosomes were transferred to independent BMDC cultures infected with *LdCen*^−/^^−^ parasites. These cultures were treated with ova peptide and co-cultured in presence of CFSE stained T cells from transgenic OT-II mice for 5 days. **(C)** Gating scheme to identify proliferation of CFSE stained CD4^+^T cells in presence of exosomes is shown. **(D)** Proliferation of CFSE stained CD4^+^ T cells in presence of exosomes isolated from uninfected DCs, **(E)** *LdWT* infection derived exosomes or **(F)** *LdCen*^−/^^−^ infection derived exosomes is shown. Proliferation in presence of exosomes derived from anti-miR-21 LNA treated cultures is also shown in these groups. **(G)** CD4^+^ OT-II cell proliferation in presence of exosomes that contain miR-21 and in presence of anti-miR-21 oligonucleotides is shown. Statistical significance between CD4^+^ T cell proliferation in presence of *LdWT* and *LdCen*^−/^^−^ infection derived exosomes 5 days post co-culture infection is shown (*t*-test; ^*^p < 0.05, ^**^p < 0.01, ^***^p < 0.001).

Recent studies showed that intracellular parasites utilize exosome mediated signaling with the host immune cells to alter host immunity (25–27) Further, in the early time points after infection, we observed that miR-21 levels were altered in bystander cells (RFP/mCherry negative population) in addition to the parasitized cells (RFP/mCherry positive population, Figures 2B–D). Consistent with this observation, at 12 h post infection, IL-12 levels were also significantly higher in *LdCen*^−/−mCherry^ parasitized cells compared to *LdWT*^*RFP*^ infection. To test if miR-21 expression has any role in mediating systemic effects, we infected murine BMDCs *in vitro* with *LdWT* or *LdCen*^−/^^−^ parasites and isolated exosomes from the culture supernatants. RT-PCR results from the exosomes precipitated from the supernatants of the infected DC cultures showed that levels of miR-21 levels were significantly higher in *LdWT* infection compared to *LdCen*^−/^^−^ (Figure 5A). In contrast, very little miR-21 was detectable above the basal level expression observed in exosomes from uninfected DCs (Figure 5A). Since our previous results established the role of miR-21 in IL-12 mediated CD4^+^ T cell proliferation, to confirm whether miR-21 enriched exosomes can affect the priming and proliferation of CD4^+^ T cells, we performed T cell co-culture experiments. To test whether exosomes enriched in miR-21 could have bystander effects, BMDCs infected with *LdCen*^−/^^−^ were cultured in presence of CFSE labeled OT-II cells. Previous studies have shown that *LdCen*^−/^^−^ infection of BMDCs results in robust proliferation of OT-II CD4^+^ T cells (13). We performed similar OT-II CD4^+^ T cell co-culture experiments in presence of exosomes enriched independently from uninfected or *LdWT* infected or *LdCen*^−/^^−^ infected BMDCs. To test the specificity of exosomal miR-21, we performed the co-culture experiments with exosomes purified from both *LdWT* and *LdCen*^−/^^−^ infection without and with treatment of LNA- anti-miR-21 oligonucleotides (Figure 5B). Dilution of CFSE staining 5 days after co-culture was measured by flow cytometric analysis as a measure of T cell proliferation (Figure 5C). Results showed that no difference in the proliferation occurred in presence of exosomes derived from uninfected DCs compared to no exosome control (Figures 5C–G). However, in presence of *LdWT* infection derived exosomes there was a significant reduction in the proliferation compared to no exosome control or in presence of exosomes derived from uninfected DCs suggesting that miR-21 enriched exosomes from *LdWT* infection exert inhibitory effects (Figure 5G, ^**^p < 0.01). Inhibition of miR-21 partially restored the OT-II cell proliferation in *LdWT* infection (Figures 5E,G, ^*^p < 0.05). No significant difference in CFSE^+^CD4^+^ T cell proliferation was observed in presence of treated and untreated *LdCen*^−/^^−^ infection derived exosomes (Figures 5F,G). Inhibition of miR-21 in *LdCen*^−/^^−^ derived exosomes did not affect the proliferation relative to no exosome, and null exosome controls (Figure 5G). These results suggest that in addition to the effects observed in the infected cells, miR-21 in the exosomes exerts strong effect on the proliferation of T cells possibly through regulation of IL-12 levels.

### *LdCen-/-* Infection Induced miR-21 Expression Alteration in Macrophages Derived From Healthy Human Blood Donors

**Figure 6:**
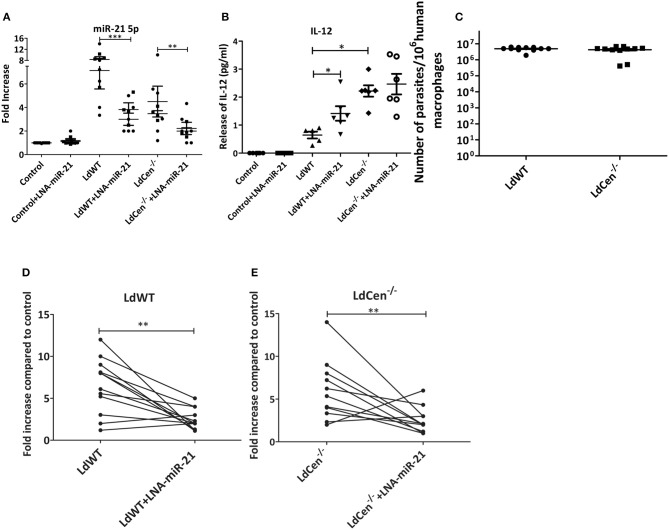
miR-21 expression mediated IL-12 regulation in human macrophages infected with *LdWT* or *LdCen*^−/^^−^ parasites. **(A)** Expression of miR-21 5p in human macrophages infected with *LdWT* or *LdCen*^−/^^−^ parasites and or treated with anti-miR-21 LNAs. Uninfected macrophages are used as controls. **(B)** Expression of IL-12 protein in the culture supernatants of macrophages infected with *LdWT* or *LdCen*^−/^^−^ parasites measured by ELISA is shown. **(C)** qRT-PCR assay showing the parasite burden in human macrophages infected with *LdWT* or *LdCen*^−/^^−^ parasites. **(D)** Expression of miR-21 in paired human macrophage cultures infected with *LdWT* parasites and treated with anti-miR-21 LNAs. **(E)** Expression of miR-21 in paired human macrophage cultures infected with *LdCen*^−/^^−^ parasites and treated with anti-miR-21 LNAs. Statistical significance between miR-21 expression or various cytokines in presence of *LdWT* and *LdCen*^−/^^−^ infection is shown (*t*-test; ^*^p < 0.05, ^**^p < 0.01, ^***^p < 0.001).

To confirm if miR-21 mediated regulation of IL-12 expression observed in murine studies is also evident in human macrophages, elutriated monocytes from US blood donors were differentiated into macrophages *in vitro*. These macrophages were infected with either *LdWT* or *LdCen*^−/−^ parasites and/or treated with LNA-anti-miR-21 oligonucleotides. RT-PCR experiments revealed that there was increase in miR-21-5p expression upon *LdWT* infection compared to *LdCen*^−/^^−^ infection and concomitant reduction in IL-12 levels in *LdWT* infection and increase in *LdCen*^−/^^−^ infection (Figures 6A,B, ^*^p < 0.05, ^**^p < 0.01, ^***^p < 0.001) similar to that observed in murine studies (Figure 2). Blocking of miR-21 resulted in a significant decrease in miR-21 levels and increase in IL-12 expression in *LdWT* infected macrophages (Figure 6B, ^*^p < 0.05). As was observed previously, such inhibition did not result in an increased IL-12 expression in *LdCen*^−/−^ infection (Figure 6B). RT-PCR assay to measure the *Leishmania* minicircle DNA showed a comparable parasite infection in the infected macrophages suggesting that the observed differences in miR-21 and IL-12 levels between *LdWT* and *LdCen*^−/−^ are not due to variation in the parasite infection level (Figure 6C). A pairwise comparison of miR-21 expression in the donor macrophages showed that in both *LdWT* and *LdCen*^−/−^ infections inhibition reduced the miR-21 expression (Figures 6D,E, ^**^p < 0.01). Taken together these data suggest that suppression of miR-21 can be a potential biomarker for *LdCen*^−/^^−^ induced immunity.

## Discussion

Live attenuated *Leishmania* parasites are investigated as potential candidate vaccines. There is a broader understanding of the key determinants of protective immunity necessary in a candidate vaccine (23). Several studies have shown the importance of IL-12 in both cutaneous and visceral leishmaniasis (23, 28–31). Protective immunity to *Leishmania* is mediated by a strong Th1 response that is critically dependent on IL-12 production by dendritic cells (32, 33). Production of IL-12 is a complex process that is regulated by multiple transcription factors that results in the production of two subunits IL-12p35 and IL-12p40 that form the functional heterodimer IL-12p70 secreted by immune cells. Therefore, the secretion of IL-12 depends on the availability of individual IL-12p35 and IL-12p40 subunits (34). The p35 and p40 subunits of IL-12 are encoded in distinct loci in the human genome (35). Whereas, the smaller p35 subunit in produced constitutively in majority of cell types, p40 subunit is transcribed more often in the context of infection or in presence of an inflammatory signal (36). Both subunits however share promoter motifs that define the transcription factor binding sites, suggesting an overall common regulatory mechanisms in producing IL-12p70 (34).

Independent studies have shown that several distinct mechanisms determine the expression of IL-12 following *Leishmania* infection. Infection with *L. donovani* has been shown to induce a differential expression pattern for IL-12-associated genes, including NF-κB factor and IFN-γ associated pathways (37). During early infection, *L. major-*infected DCs exhibited a distinct type-I IFN-associated transcriptomic signature, including the upregulation of IRF2, IRF9, STAT1/2*, and* IFNAR (38). Differences in *L. major* and *L. donovani* infections with respect to expression of *IRF2, IRF7*, and *IFIT5* was also reported analogous to the IL*-*12p40 gene expression elicited by these two parasite species (38). Similarly, in *L. major* infections, ligation of CD40-CD40L, and macrophage antigen-1 (Mac-1)-CD40L interaction have been shown to induce IL-12 production (39). In this study we explored an additional mechanism of regulation of IL-12 by miR-21 that is used by the *Leishmania* parasite.

Previous studies in allergic airway inflammation have shown that the microRNA miR-21 targets the 3′UTR of the IL-12 p35 and expression of miR-21 was shown to result in the loss of I-12p35 transcript (22). Studies in miR-21^−/−^ mice using Ova peptide stimulation showed dysregulation of IL-12/IFN-γ pathway upon transcriptome analysis, that resulted in altered T cell polarization implying the role of miR-21 in regulating the expression of IL-12p35 (21). Our results first showing the upregulation of miR-21 in virulent *L. donovani* infection compared to *LdCen*^−/−^ infection represents a novel mechanism by which IL-12 production is regulated in the parasitic infections. Stimulation of IL-12 production upon inhibition of miR-21 both *in vitro* and *in vivo* shows the specificity of the miR-21 and IL-12 production. Further, lack of induction of miR-21 in *LdCen*^−/−^ infection compared to *LdWT* infection may represent an immunological mechanism orchestrated by *LdCen*^−/^^−^ parasites that results in increased IL-12 production. Early heightened induction of IL-12 has been shown to be required for the induction of protective immunity in several Leishmania studies (28, 40). Thus, the IL-12 expression observed in *LdCen*^−/^^−^ parasite infection suggests that miR-21 mediated regulation might be a key step in promoting strong protective immune response.

In addition to altering the miR-21 expression in the infected cells, our results also showed that exosomes released from *LdCen*^−/^^−^ infection contained reduced miR-21 levels compared to *LdWT* infection, representing a systemic effect propagated by the exosomes derived from *LdCen*^−/^^−^ infected cells. Our results on the abundance of miR-21 in the exosomes secreted by the *LdWT* infected DCs exerting anti-inflammatory effects show that the induction of miR-21 can lead to systemic effects by contact-independent mechanisms. Consistent with this result, bystander APCs with *LdWT* infection identified in our *in vivo* experiments also showed reduced IL-12 in our experiments at early time points indicating that exosome mediated miR-21 may be involved in such reduction. In other studies plasma exosome miR-21 is known to indicate breast cancer (41). Human macrophages infected with BCG also secrete exosomes containing miR-21 (42). Similar effects mediated by exosomes on IL-12 and TNF were reported previously (43). Our results with exosomes derived from *LdWT* infected DCs on the T cell proliferation suggest that miR-21 enriched in the exosomes could be a principal mechanism by which IL-12 regulation could be achieved overall. Our study shows that redundant mechanisms might exist to achieve that outcome including miR-21 mediated degradation of IL-12 mRNA. The exosome mediated IL-12 regulation is likely to occur through miR-21 mediated effects. This argument is supported by our findings that inhibition of miR-21 by anti-sense oligos in our *in vitro* experiments and by LNA based inhibitors in the *in vivo* experiments showed a significant induction of IL-12. The central question as to what causes miR-21 expression to be subdued in *LdCen*^−/−^ parasite infection relative to *LdWT* infection remains to be investigated. The attenuated virulence of *LdCen*^−/−^ parasites might lead to such overall effect. This hypothesis is supported by our results with killed *LdCen*^−/−^ that failed to suppress miR-21 induction suggesting that factors regulating miR-21 suppression are produced only by the live *LdCen*^−/^^−^ parasites.

In addition to IL-12, miR-21 is shown to target PDCD4 and IL10 (44). Quantitative RT-PCR results with *LdWT* and *LdCen*^−/^^−^ infected murine DCS showed no significant difference in the PDCD4 levels (data not shown). Further, our results showed that IL-10 levels were not significantly altered between *LdWT* and *LdCen*^−/^^−^ infections upon blocking miR-21 indicating that specifically IL-12 regulation through miR-21 occurred in *LdWT* and *LdCen*^−/^^−^ parasite infections. Additional role for exosomal miR-21 has been described in inducing alternative activation of macrophages (M2 type) (45). As the live attenuated *Leishmania* parasites such as *LdCen*^−/^^−^ are tested in pre-clinical studies as candidate vaccines, our studies identify a key characteristic that may indicate a critical immune regulatory mechanism that is necessary for protective immunity. In addition to CD40 mediated IL-12 expression observed in our previous studies (13), our current study represents a novel mechanism by which IL-12 expression is regulated. Previous studies comparing the expression of miR-21 showed that wild type *L. donovani* infection induces higher levels compared to *L major* infection suggesting that differences might exist between different species of *Leishmania* (46). Our results showed that in addition to miR-21, miR-3651, and miR-3679 were also enriched in *LdWT* compared to *LdCen*^−/−^ infection. Although miR-3651 and miR-3679 are reported in the literature as prognostic biomarkers for myesthnia gravis, sepsis or squamous cell carcinoma (47–50) their role in gene expression regulation has not been studied.

Taken together, our results show that miR21 is a key determinant of IL-12 production following *L. donovani* infection and that live attenuated parasite infection such as *LdCen*^−/−^ causing suppression of miR-21 is a novel finding and suggests that infection with attenuated parasites produces controlled inflammation thus leading to protective immunity.

## Materials and Methods

### Parasites and Animals

Five to six weeks old female C57Bl/6 mice were obtained from the Charles River Laboratories. The animal procedures and experiments described were approved by FDA's Animal Care and Use Committee (Study 1995–26, updated and re-approved 8/18/2016). The animal program is fully compliant with the US PHS Policy on Humane Care and Use of Laboratory Animals and standards for full accreditation by AAALAC International. The wild type *L*. *donovani* (*LdWT*) (MHOM/SD/62/1S) maintained in golden Syrian hamsters and centrin gene deleted (*LdCen*^−/−^*)* line of *L*. *donovani* were used in the experiments. Red fluorescent protein expressing *LdWT*^*RFP*^ and mCherry protein expressing *LdCen*^−/−^ (*LdCen*^−/−mCherry^) parasites were used to sort select infected DCs from C57Bl/6 mice. *LdWT* and *LdCen*^−/−^ parasites secreting 2W epitope (EAWGALANWAVDSA) fused to *L. donovani* 3′ Nucleotidase/Nuclease protein lacking the N' terminus membrane anchoring domain, were used to enrich epitope specific CD4^+^ T cells. The recombinant *LdWT* and *LdCen*^−/^^−^ parasites expressing fluorescent or chimeric proteins have been described previously (11, 24). The 2W-PE tetramers (2W1S:I-A^b^-streptavidin-phycoerythrin) used to enrich the CD4T cells were obtained from NIH tetramer core facility in Emory Vaccine Center, Atlanta, GA.

### Human Monocytes

Human elutriated monocytes obtained from NIH blood bank from healthy US blood donors. Only monocytes that tested CMV negative were used in this study. Monocytes were resuspended at 1.8 × 10^5^ cells/ml in RPMI medium containing 10% FBS and human macrophage colony stimulating factor (20 ng/ml, ProSpec), plated in a volume of 0.5 ml in eight-chamber Lab-Tek tissue culture slides (Miles Laboratories) and incubated for 7 days for differentiation into macrophages.

### Macrophage Infection

The differentiated macrophages were infected with stationary phase *LdWT* or *LdCen*^−/−^ promastigotes (10:1 parasite-to-macrophage ratio). After incubation for 6 h at 37°C in 5% CO_2_, the free extracellular parasites were removed by RPMI washes and the cultures were incubated in macrophage culture medium for an additional 24 h. The culture medium was removed and total RNA was extracted from the infected macrophages using Purelink Ambion kit. RNAseq was performed at the core facility of Center for Biologics Evaluation and Research, FDA.

### *In vitro* Blocking of miR-21

Dendritic cells were cultured *in vitro* from bone marrow progenitors. Bone marrow aspirates from C57Bl/6 mice were cultured with complete RPMI medium supplemented with 10% (v/v) fetal bovine serum (FBS) and 20 ng/mL murine GM-CSF (Peprotech) and murine IL-4 (Peprotech) for 7 days at 37°C and 5% CO_2_ to obtain CD11c^+^ DCs. DCs were infected with either *LdWT* or *LdCen*^−/−^ parasites for 6 h (10:1 parasite to DC ratio). Free extracellular parasites were removed by extensive washes with complete RPMI. Infected DCs were incubated with anti-miR-21 oligos packaged in mouse DC derived exosomes (XMIR-21, System Biosciences) for 24 h. Total RNA was isolated from the infected XMIR-21 treated DCs and used in RT-PCR assays.

### Expression of miR-21 *in vivo* in LdWT or *LdCen^−/^^−^* Infected DCs

C57Bl/6 mice were infected intravenously with fluorescent live or killed *LdWT*^*RFP*^ or *LdCen*^−/−−mCherry^ parasites. Spleens were collected from the mice 24 h after the infection, homogenized and single cell suspensions were prepared after ACK lysis. DCs were isolated from the cell suspension using Pan-Dendritic cell isolation kit (Miltenyi Biotec). Parasitized DCs were sort selected based on the RFP or mCherry fluorescence (FACSAria II, BD). RNA isolated from the parasitized DCs was used in a qRT-PCR to detect the expression of miR-21.

### RT-PCR

Total RNA was extracted from the parasitized DCs using RNAqueous Microkit (Ambion). Total RNA was reverse transcribed into cDNA using random hexamers by a high-capacity cDNA reverse transcription kit (Applied Biosystems). Gene expressions were determined by TaqManGene Expression Master Mix and pre-made TaqMan Gene Expression assays (Applied Biosystems) using a CFX96 Touch real-time system (Bio-Rad). The data were analyzed with CFX Manager soft-ware. The TaqMan Gene Expression Assay ID (Applied Biosystems) of different primers used was as follows: miR-21 (Mm00434174); and GAPDH (Mm99999915). Expression values were determined by the 2-^ΔΔCt^ method where samples were normalized to GAPDH expression.

### CD4^+^ T Cell Proliferation

CD4^+^ T cell proliferation was measured in mice infected with *LdWT* or *LdCen*^−/−^ followed by treatment with miR-21 inhibitor LNAs (10 μg/dose). At each experimental time point post infection spleens, inguinal, axillary, cervical, submandibular, and parotid lymph nodes were collected. Single cell suspensions from these organs were used to enrich for 2W specific CD4^+^ T cells using tetramer reagents on LS columns (Miltenyi). Enriched cells were labeled and analyzed using flow cytometry.

### CD4^+^ T Cell Proliferation in Presence of Exosomes

Bone marrow aspirates from C57Bl/6 mice were cultured with complete RPMI medium supplemented with 10% (v/v) fetal bovine serum (FBS) and 20 ng/mL murine GM-CSF and IL-4 (Peprotech) for 7 days at 37°C and 5% CO_2_ to obtain CD11c^+^ DCs. BMDCs were infected with either *LdWT* or *LdCen*^−/−^ parasites for 6 h (10:1 parasite to DC ratio). The parasite infections were performed either in presence or absence of anti-miR-21 oligonucleotides for 24 h in complete RPMI medium containing 10% exosome-free FBS (System Biosciences). The exosomes released by the uninfected, parasite infected and/or anti-miR-21 treated BMDC cultures were pelleted using ExoQuickTC reagent (System Biosciences). In parallel, another set of BMDC cultures were pulsed with 2 μg/ml OVA peptide (323–339, AnaSpec) for 4 h and infected with *LdCen*^−/−^ parasites. T cells isolated from a transgenic OT-II mouse using Pan-T cell isolation kit (Miltenyi Biotec) were labeled with CFSE and co-cultured with Ova pulsed, *LdCen*^−/−^ infected BMDCs above in presence or absence of exosomes isolated as described above. Flow cytometric analysis was performed to measure the proliferation of CFSE labeled OT-II T cells after 5 days of co-culture in presence or absence of exosomes. Cells were blocked at 4°C with rat anti-mouse CD16/32 and stained with surface markers on ice with anti-B220, anti-CD11b, anti-CD11c, and anti-F4/80 all conjugated with eFluor 450; Alexa Flour 700-anti-CD3, BV-785 anti-CD4, and BV-650 anti-CD8α. Live cells were selected from Live/dead aqua staining. Cells were then analyzed on an LSR Fortessa (Becton Dickinson). Data were analyzed with FlowJo software v10 (TreeStar).

### Flow Cytometry

Tetramer staining was performed following the published protocol (24). Single cell suspensions were prepared from spleens and lymph nodes isolated from the infected mice. Cell suspensions were treated with 3 ml of ACK lysis buffer (Lonza) for 5 min at room temperature. Cells were stained with 2W-PE tetramers for 1 h in the dark at room temperature. The tetramer coated cells were labeled with anti-PE magnetic beads for 30 min on ice. 2W epitope specific CD4^+^ T cells were enriched on MACS LS magnetic columns. Cells were blocked at 4°C with rat anti-mouse CD16/32 and stained with surface markers on ice with anti-B220 (eBioscience, Clone: RA3-6B2, Cat# 48-0452-82), anti-CD11b (eBioscience, Clone: M1/70, Cat# 48-0112-82), anti-CD11c (eBioscience, Clone:BM8, Cat# 48-0114-82), and anti-F4/80 (eBioscience, Clone:BM8, Cat# 48-4801-82) all conjugated with eFluor 450 (Dump); Alexa Flour 700-anti-CD3 (Biolegend, Clone: H57-597, Cat# 109224), BV-785 anti-CD4 (Biolegend, Clone:RM4-5, Cat# 100552), BV-650 anti-CD8α (Biolegend, Clone: 53-6.7, Cat# 100742), FITC-anti-CD44 (Biolegend, Clone:IM7, Cat# 103006), PerCP-Cy5.5 CD25 (BD, Clone: PC61, Cat# 563866), BV-605 anti-CD62L(eBioscience, Clone:MEL-14, Cat# 83-0621-42), APC anti-IL7R (eBioscience, Clone:A7R34, Cat# 50-1271-82). Live cells were selected from Live/dead aqua staining. Cells were then analyzed on an LSR Fortessa (Becton Dickinson). Data were analyzed with FlowJo software v10 (TreeStar).

### ELISA to Measure Murine IL-12, TNF, and IL-10

Murine BMDCs were prepared as described (13) and were infected with *LdWT* or *LdCen*^−/−^ parasites with or without LNA treatment. The culture supernatants were analyzed by ELISA for indicated cytokines. The conditioned media of DC cultures were assayed for mouse cytokines via sandwich ELISA kit (eBioscience, IFN, IL-10, TNF, and IL-12). The assay was performed according to the manufacturer's instructions.

### Parasite Load Determination by Quantitative PCR

The parasite load was determined in the infected dendritic cells and human macrophages as previously described (51). Briefly, infected DCs/infected human macrophage samples were lysed for DNA purification by using DNeasy Blood & Tissue kits (Qiagen). Seventy nanograms of sample DNA was used as a template in a Taqman-based quantitative PCR. The target DNA was amplified from a kinetoplast minicircle DNA of the parasite using the following sequence of primers: *Leishmania* forward primer 5′- CTATTTTACACCAACCCCCAGT-3′ *Leishmania* reverse primer 5′-GGGTAGGGGCGTTCTGCGAAA-3′ with the addition of a fluorescent probe 5′-RAAARKKVRTRCA GAAAYCCCGT-3′ for detection. A Black Hole Quencher moiety was coupled to the 3′ end and Calfluor Red was coupled to a C6 linker at the 5′ end. The fluorescent Leishmania probe (5′-RAAARKKVRTRCAGAAAYCCCGT-3′) was added to the reaction mixture at a final concentration of 1.5 pmols/μl. Cycling parameters were as follows: preheat at 95°C for 180 s and then 40 two-step cycles of 95°C for 10 s and 55°C for 30 s. To determine the number of *Leishmania* cells that were represented by a given cycle threshold (Ct) value, a standard curve was constructed by purification of DNA from naïve mice DCs/elutriated human macrophages spiked with a known number of parasites.

### Statistical Analysis

Statistical analysis of differences between mean values of groups was determined by parametric unpaired two-tailed Student *t*-test and non-parametric Mann–Whitney test using GraphPad Prism 5.0 software. A *p* < 0.05 was considered significant.

## Data Availability Statement

The datasets generated for this study are available on request to the corresponding author.

## Ethics Statement

The animal study was reviewed and approved by FDA's Animal Care and Use Committee.

## Author Contributions

SG and HN conceived the project. SG, AS, PB, and NI performed the experiments. SG, PB, SM, and HN analyzed the data. SG, PB, and HN wrote the manuscript.

### Conflict of Interest

The authors declare that the research was conducted in the absence of any commercial or financial relationships that could be construed as a potential conflict of interest.

## Supplementary Material

Supplementary Figure 1 Parasite infectivity of human macrophages. Human macrophages from four independent blood donors were infected with stationary phase *LdWT* or *LdCen*^−/^^−^ parasite cultures. Twenty four hours post infection, the infected macrophages were fixed and stained with diff-quik staining reagents. The percentage of infected macrophages were counted in triplicate and expressed as % infected macrophages.

Supplementary Figure 2 Secretion of IL-10 and IFN following blocking of miR-21. **(A)** Expression of IL-10 protein in the culture supernatants of macrophages infected with *LdWT* or *LdCen*^−/^^−^ parasites measured by ELISA is shown. **(B)** Expression of IFN-γ protein in the culture supernatants of macrophages infected with *LdWT* or *LdCen*^−/^^−^ parasites measured by ELISA is shown.

Supplementary Table 1 Enrichment of microRNAs and non-coding RNAs in *Leishmania* infected human macrophages. Human macrophages from four independent blood donors were infected with stationary phase *LdWT* or *LdCen*^−/^^−^ parasite cultures. RNA was isolated from the infected macrophages 24 h post infection and subjected to small RNAseq reaction. Identities of the small RNAs enriched in uninfected control relative to *LdCen*^−/^^−^ infection (Up UI vs. Cen tab in the excel sheets), and those enriched in *LdWT* infected macrophages relative to *LdCen*^−/^^−^ infection (Up WT vs. Centrin tab in the excel sheets) are shown.
