# Supplementary figures and images for: miR-21 Expression Determines the Early Vaccine Immunity Induced by LdCen−/− Immunization
Source: Front Immunol. 2019 Sep 24;10:2273. doi: 10.3389/fimmu.2019.02273 (PMC6769120; doi:10.3389/fimmu.2019.02273)

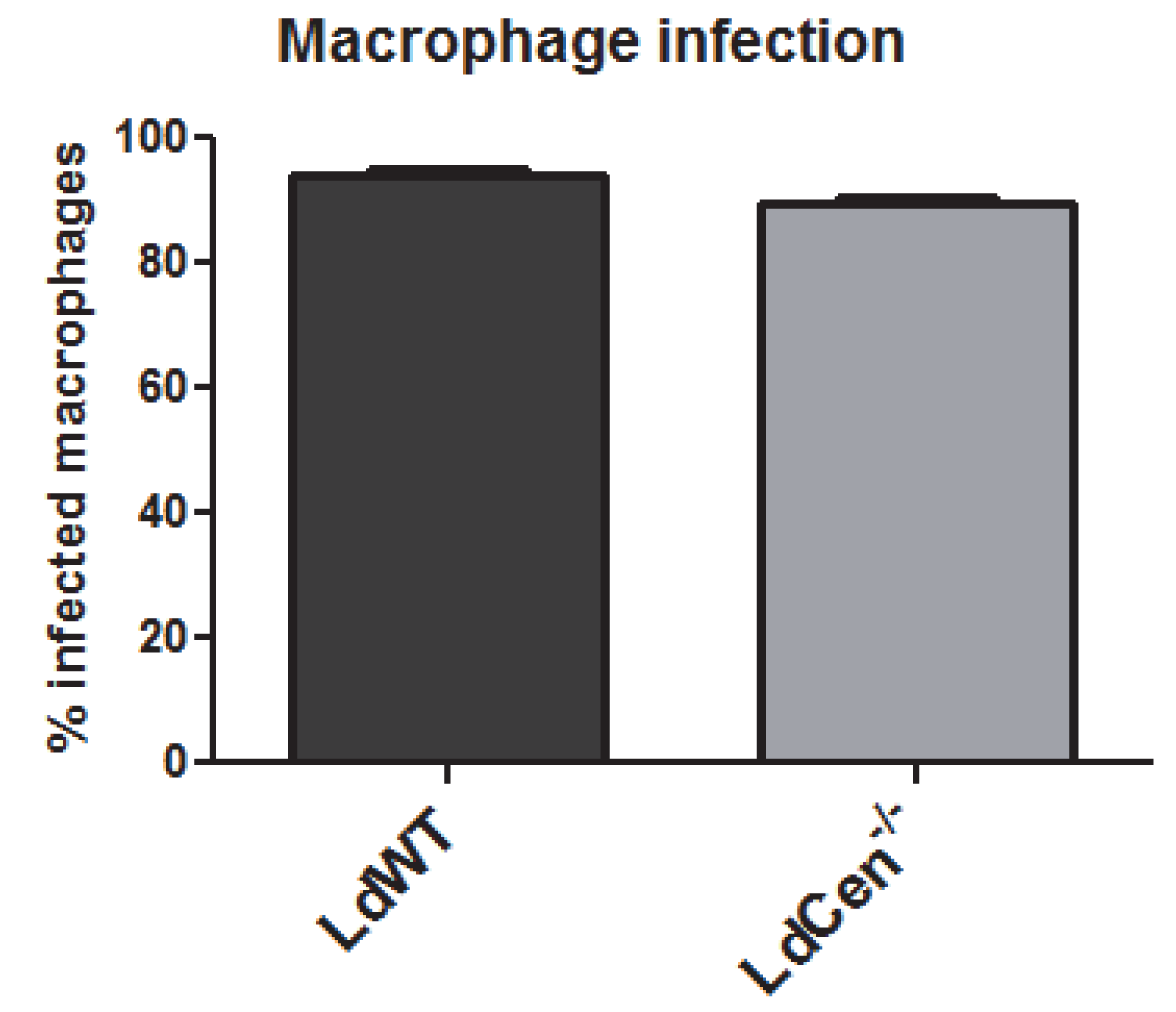

Supplement: Supplementary Figure 1 — Parasite infectivity of human macrophages. Human macrophages from four independent blood donors were infected with stationary phase LdWT or LdCen−/− parasite cultures. Twenty four hours post infection, the infected macrophages were fixed and stained with diff-quik staining reagents. The percentage of infected macrophages were counted in triplicate and expressed as % infected macrophages. [file Image_1.TIF]

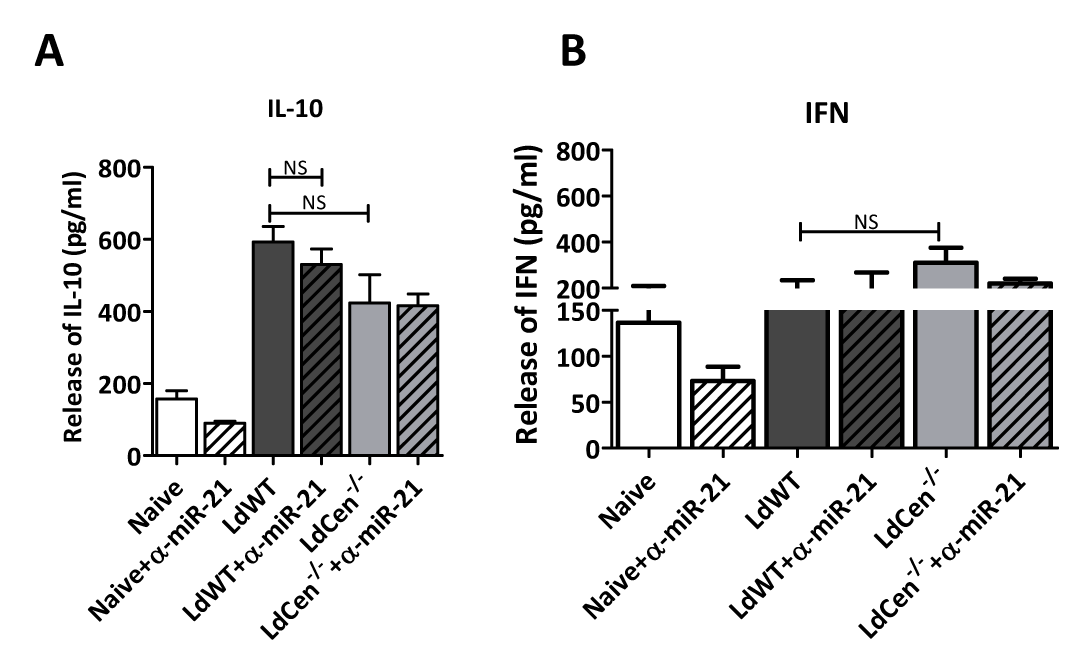

Supplement: Supplementary Figure 2 — Secretion of IL-10 and IFN following blocking of miR-21. (A) Expression of IL-10 protein in the culture supernatants of macrophages infected with LdWT or LdCen−/− parasites measured by ELISA is shown. (B) Expression of IFN-γ protein in the culture supernatants of macrophages infected with LdWT or LdCen−/− parasites measured by ELISA is shown. [file Image_2.TIF]
